# Bacterial lipopolysaccharide-induced endothelial activation and dysfunction: a new predictive and therapeutic paradigm for sepsis

**DOI:** 10.1186/s40001-023-01301-5

**Published:** 2023-09-12

**Authors:** Min Wang, Jun Feng, Daixing Zhou, Junshuai Wang

**Affiliations:** 1 Department of Emergency Medicine, Tongji Hospital, Tongji Medical College, Huazhong University of Science and Technology, 1095 JieFang Avenue, Wuhan, 430030 Hubei People’s Republic of China; 2 Department of Critical Care Medicine, Tongji Hospital, Tongji Medical College, Huazhong University of Science and Technology, 1095 JieFang Avenue, Wuhan, 430030 Hubei People’s Republic of China

**Keywords:** Lipopolysaccharide, TLR4, Endothelial cells, Sepsis

## Abstract

**Background:**

Lipopolysaccharide, a highly potent endotoxin responsible for severe sepsis, is the major constituent of the outer membrane of gram-negative bacteria. Endothelial cells participate in both innate and adaptive immune responses as the first cell types to detect lipopolysaccharide or other foreign debris in the bloodstream. Endothelial cells are able to recognize the presence of LPS and recruit specific adaptor proteins to the membrane domains of TLR4, thereby initiating an intracellular signaling cascade. However, lipopolysaccharide binding to endothelial cells induces endothelial activation and even damage, manifested by the expression of proinflammatory cytokines and adhesion molecules that lead to sepsis.

**Main findings:**

LPS is involved in both local and systemic inflammation, activating both innate and adaptive immunity. Translocation of lipopolysaccharide into the circulation causes endotoxemia. Endothelial dysfunction, including exaggerated inflammation, coagulopathy and vascular leakage, may play a central role in the dysregulated host response and pathogenesis of sepsis. By discussing the many strategies used to treat sepsis, this review attempts to provide an overview of how lipopolysaccharide induces the ever more complex syndrome of sepsis and the potential for the development of novel sepsis therapeutics.

**Conclusions:**

To reduce patient morbidity and mortality, preservation of endothelial function would be central to the management of sepsis.

**Graphical Abstract:**

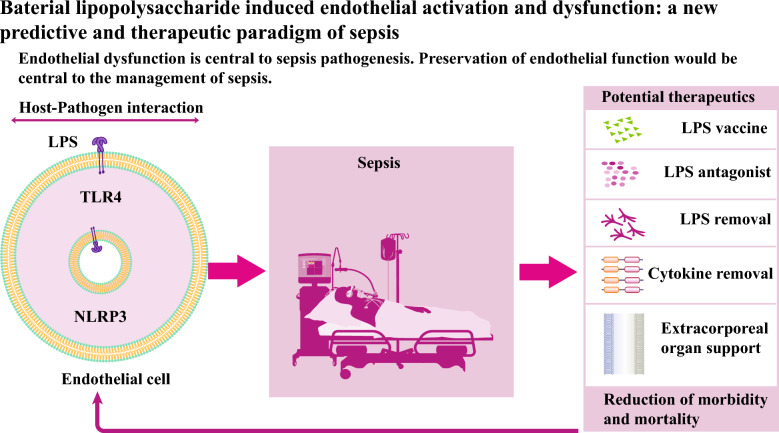

## Introduction

Sepsis is a multistep and complex pathophysiological process involving an inappropriate host inflammatory and immune response that can lead to multiple organ failure. To date, bacterial sepsis continues to be the leading cause of death worldwide [1]. The virulence of pathogenic bacteria is highly dependent on the production of toxins that can invade the target eukaryotic cells. They are either structural components of the bacterial cell wall (endotoxin) or synthesized and secreted proteins (exotoxin) [2, 3]. Accumulating evidence suggests that the critical events underlying the pathogenesis of dramatic dysregulation during sepsis are the systemic dissemination of microbial toxins rather than bacteraemia itself [4, 5]. For example, both lipopolysaccharide (LPS, also known as endotoxins) from gram-negative pathogens and peptidoglycan and lipoteichoic acid from gram-positive pathogens can activate the host immune response, leading to dysregulated inflammation, microcirculatory dysfunction and death [6, 7]. LPS is detected in the bloodstream not only in gram-negative infections but also in gram-positive and fungal infections, probably because of a sepsis-related breakdown of the intestinal barrier [8, 9]. LPS is recognized as a highly pathogenic endotoxin responsible for organ dysfunction in sepsis [10, 11]. Once inside the host, LPS activates the innate immunity of the host, stimulating humoral and cellular responses that lead to inflammation and toxicity [12]. Therefore, LPS can induce specific pathologic changes in multiple organs, leading to poor outcomes [13, 14]. As the major endotoxin of pathogenic gram-negative bacteria, the virulence of LPS has been extensively studied [15, 16].

Endothelial cells (ECs) form a critical thin endothelial monolayer lining the innermost surface of blood vessels, providing an interface between blood and tissue [17, 18]. In addition to their involvement in physiological processes, ECs play important roles in both innate and adaptive immune responses, including migration, proinflammation, phagocytosis, antigen presentation, sensing, cytokine secretion, immunomodulation, immunosuppression, and plasticity [19]. An important advance in our understanding of sepsis has been the identification of markers of exaggerated inflammation, endothelial dysfunction and coagulopathy as indicators of impaired organ function [20, 21]. While the extent of endothelial dysfunction due to direct effects of LPS on ECs after EC activationversus indirect effects secondary to the release of inflammatory mediators from immune cells remains controversial, many studies have focused on the pivotal role of ECs in the pathogenesis of sepsis [22]. This review is intended to give an overview on the pathological effects and of immune response of LPS on the endothelial cells. In addition, the complex pathophysiology of sepsis and the lack of effectiveness of current therapies will promote the development of novel therapies guided by biomarkers predictive of clinical response.

## The molecular structure determines virulence

Approximately 2 × 10^6^ molecules of LPS cover nearly 75% of the bacterial surface under exponential growth conditions [23]. LPS consists of three distinct domains, including a glycolipid moiety called lipid A, a polysaccharide, and an oligosaccharide core, which are covalently bound together and differ in genetics, biosynthesis, biology, and chemistry [8, 18]. The outer membrane anchor, lipid A, consists of a glucosamine disaccharide core and six fatty acid acyl chains, which is responsible for the proinflammatory properties of LPS [24]. It has been shown that the basic architecture of hexaacylated, bisphosphorylated lipid A alone is capable of eliciting responses in ECs that are identical to those induced by LPS [25]. Therefore, agents that specifically target the LPS lipid A component are effective in inhibiting endothelial activation and ameliorating vascular complications in endotoxin shock models [26]. The *O*-antigen consists of domains of repetitive sugar oligomers, is highly flexible, and does not appear to contribute to LPS pathogenicity. However, *O*-polysaccharides are critical for the evasion of bacteria from host immune effectors. The stability and permeability of the outer membrane is thought to depend on the LPS core. The inner core is necessary for gram-negative virulence. A critical part of LPS pathogenicity is the innermost part of the core oligosaccharide [27, 28]. The negatively charged groups in the inner core can form multiple bonds with TLR4/MD-2. This interaction is critical for the dimerization of the TLR4/MD2–LPS complex [29, 30]. In addition, the O-chain triggers an effective humoral immune response to induce substantial secretion of antibodies into the blood circulation [31]. As a result, LPS can trigger potential inflammatory reactions, and even low blood concentrations can cause serious sepsis and complications in humans [10, 32]. Various extracellular and intracellular pathways are involved in LPS sensing, and non-canonical activation of caspase-mediated pyroptosis is thought to play an important role in the pathophysiology of sepsis. LPS induces specific pathological changes in several organs, contributing to poor outcomes [30]. In fact, it is the delicate structure of LPS that gives it its specific virulence.

**Fig. 1:**
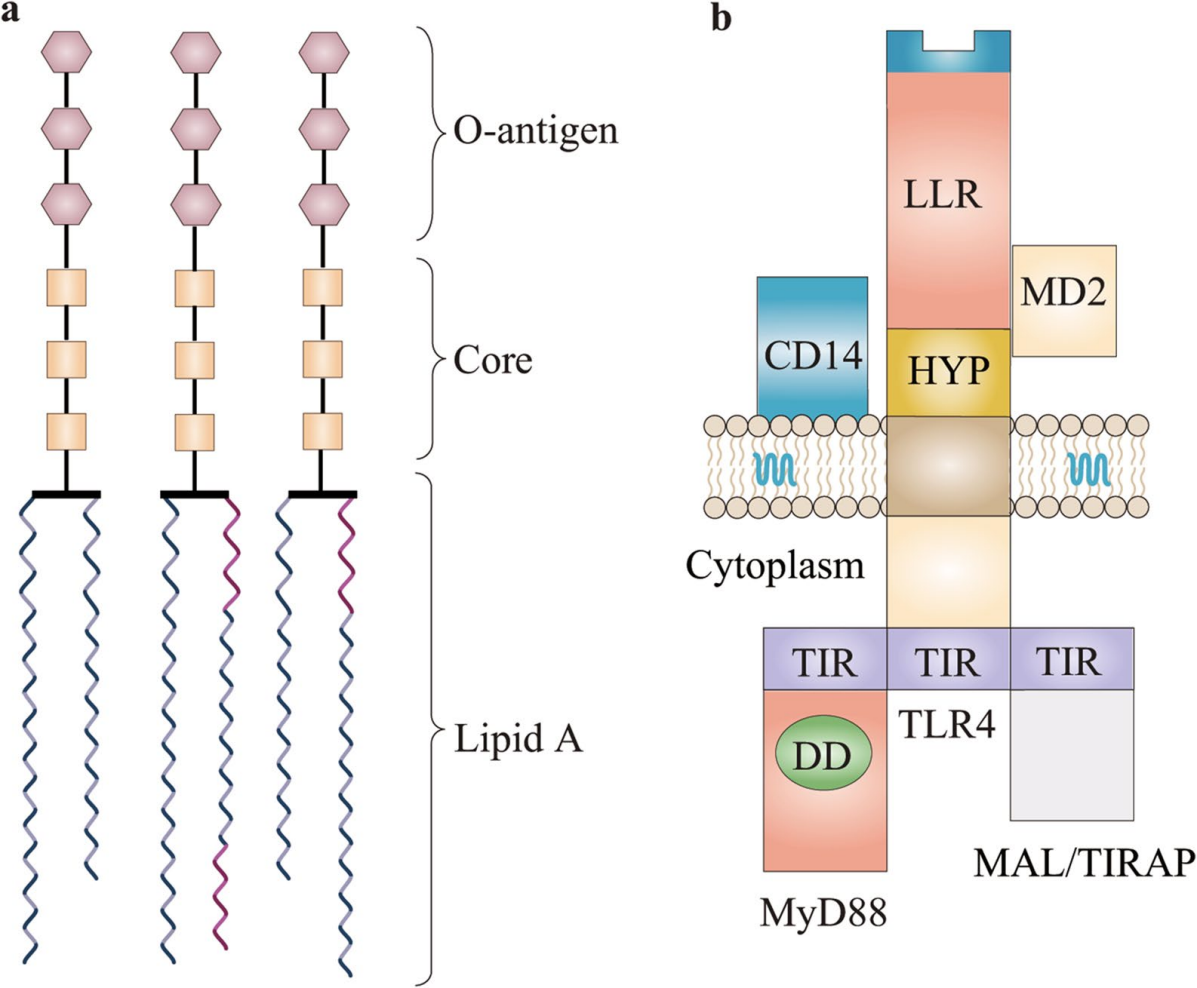
Schematic structure of lipopolysaccharide (LPS) and the TLR4 receptor complex. **a** LPS is composed of lipid A (endotoxin), core oligosaccharide and O-antigen. **b** Components of the TLR4–MD2–CD14 receptor complex. Different TLR4 regions are shown: leucine-rich repeats (LRR), a hypervariable region (HYP) and the intracellular TIR domain. myeloid differentiation factor 88 (MyD88), MyD88 adapter-like protein/Toll receptor IL-1 receptor domain containing adapter protein (MAL/TIRAP). LRR, leucine-rich repeats; DD, death domain; TIR, Toll receptor IL-1 receptor domain

The major advance has been the identification of the membrane protein TLR4 as the Lipid A receptor of eukaryotic cells. TLR4 belongs to a family of pattern recognition receptors (PRRs) that mediate inflammatory responses that are always beneficial in controlling local inflammation but can become deleterious in an overwhelmed systemic response [25]. The inner core of LPS is able to form covalent bonds with TLR4/MD2, an interaction that is critical for dimerizing the TLR4/MD2–LPS complex. Lipid A, which binds to the TLR4/MD2 complex and triggers the biosynthesis of major inflammatory mediators, such as TNF-α, IL1-β and IL-6, plays the most prominent role in LPS pathogenicity during the host–bacteria interaction. It promotes the adaptive immune response through enhancement of the antigen presentation capacity of host cells, such as macrophages and monocytes [29]. The critical role of acyl side chains in TLR4 signaling is suggested by further structural and functional analysis of lipid A signaling [33, 34]. Despite the structural variability in different bacterial species, the structural pattern of the LPS–TLR4 complex could provide a perspective for drug synthesis and vaccine development. The schematic molecular structure of LPS and components of the TLR4–MD2–CD14 receptor complex are shown in Fig. 1.

## Signaling pathways of LPS in endothelial cells

There is no doubt that LPS is an important mediator of sepsis during infection with gram-negative bacteria and after absorption from the intestinal infection. LPS is able to induce an inflammatory response through both intracellular and extracellular pathways. TLR4 plays a key role in the initiation of the innate immune response and its activation with LPS has been implicated in chronic and acute inflammatory diseases. Among the diseases caused by the abnormal activation of TLR4, sepsis is the most dangerous, because it is a life-threatening acute inflammatory condition of the system for which there is still a lack of specific pharmacological treatment [35]. Extracellularly, ECs recognize LPS presence through a receptor complex containing at least three essential cell surface components: CD14, TLR4 and MD-2 [36]. TLR4, in cooperation with MD-2, mediates the innate cellular response to LPS, as described above. CD14 is important for the activation of TLR4 by the smooth form of LPS [37]. LPS binding protein (LBP) can significantly increase the binding of LPS to TLR4 [38, 39]. ECs then recognize the presence of LPS and recruit specific adaptor proteins to TLR4 membrane domains, initiating an intracellular signaling cascade.

At least five levels of receptor- and ligand-dependent specificity of the LPS signal transduction pathway have been identified. At the first level, LPS binds to LBP in the classical recognition between LPS and ECs. The LPS–LBP complex then binds to the TLR4–MD2–CD14 receptor complex and initiates a signaling cascade [40]. In the second level, the activated TLR4–MD2–CD14 complex initiates two subsequent signaling pathways: (i) MyD88-dependent and (ii) MyD88-independent [41]. In the MyD88-dependent pathway, MyD88 recruits IL-1 receptor-associated kinase (IRAK) to TLR4, leading to the activation of two distinct signaling pathways, ultimately leading to the activation of JNK and NF-κB. In the MyD88-independent pathway, LPS stimulation activates the transcription factor interferon regulatory factor 3 (IRF-3), thereby inducing IFN-β for the subsequent induction of several IFN-inducible genes. These ligand-specific signal transduction events constitute the third level [42]. The fourth level is the ubiquitination of TRAF-6, which triggers its oligomerization and the assembly and activation of a multiprotein complex. This complex activates Iκ kinases and the mitogen-activated protein kinase (MAPK) family. In the fifth step, the MAP kinase family subsequently transactivates NF-κB transcription and further modulates expression by directly interacting with promoters of proinflammatory genes [40, 43]. Finally, LPS binding to the EC surface induces endothelial activation, as evidenced by proinflammatory cytokine and adhesion molecule expression and endothelial damage.

**Fig. 2:**
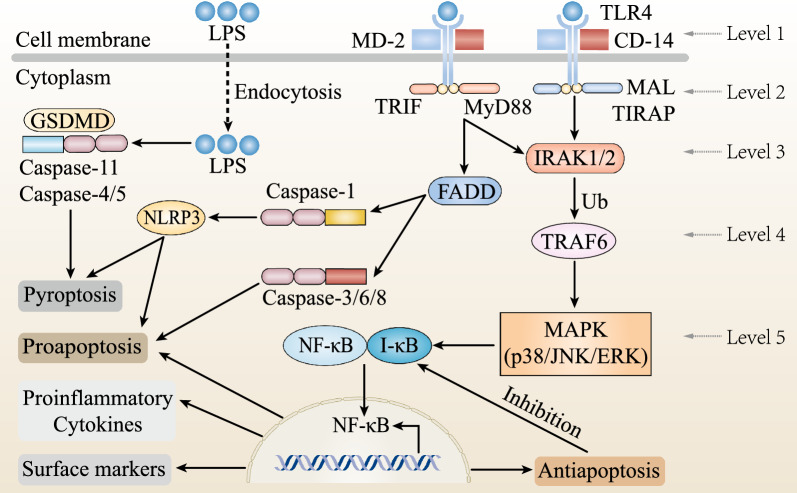
Proposed signal transduction pathways in endothelial cells in response to LPS exposure. The extracellular pathway, which represents the canonical LPS signaling cascade, is initiated by the recognition of LPS by the TLR4–MD2–CD14 complex. At least five levels of receptor- and ligand-dependent specificity are currently recognized following activation of TLR4, which have been summarized in the text. On the other hand, LPS can be internalized by endothelial cells through endocytosis. Intracellular LPS recognition triggers caspase-11 activation, NLRP3 inflammasome activation and pyroptosis. NF-κB activation in turn results in the transcriptional induction of proinflammatory cytokines (IL-6, IL-8, and IL-1), proapoptotic cytokines (TNFR1, Fas and DR3/4/5), antiapoptotic cytokines (cIAPs, FLIP, A1 and A20) and surface markers (E-selectin, ICAM-1, VCAM-1 and TF)

However, TLR4 inhibitors have not been as effective as expected in treating sepsis, and the clinical effect of endotoxin adsorption remains to be determined, suggesting that another TLR4-independent pathway may be more critical for LPS-induced endotoxemia and endotoxic shock [13]. This led to the identification of the intracellular LPS sensing and signaling pathway. This pathway induces sepsis when activated by cytosolic LPS without the requirement of TLR4, termed the noncanonical inflammasome [44]. LPS binding to endothelial adhesion molecules has been shown to mediate the membrane translocation of bacteria [45, 46]. The noncanonical inflammasome, which recognizes cytosolic LPS, can induce lethal sepsis by binding of cytosolic LPS to caspases, leading to pyroptosis and activation of the NLRP3 inflammasome followed by secretion of IL-1β and IL-18 [47, 48]. Intracellular recognition of LPS triggers caspase-11 and caspase-4/5 in mice, which leads to pyroptotic cell death [49]. Pyroptosis is characterized by severe cell membrane damage leading to passive intracellular inflammatory release [50]. Caspase-11-dependent cleavage of gasdermin D has been implicated as a multifaceted linker among LPS-induced caspase activation, NLRP3 inflammasome activation, and pyroptosis [39, 51]. Consistent with these studies, the NF-κB, MAPK, and PI3K pathways, which are common to both intracellular and extracellular pathways, are activated as key functional players in the effects of intracellular LPS [43, 52]. Finally, cytoplasmic LPS activates caspase-11-dependent pyroptosis, leading to endotoxemia-related lethality. In part, the TLR4-independent LPS sensing mechanism may be more important in the development of sepsis than the TLR4-dependent LPS sensing mechanism [47, 53]. With potential clinical implications for the treatment of sepsis, this novel intracellular LPS sensing pathway provides a new paradigm for the development of LPS-induced endotoxemia [54]. Figure 2 illustrates the signal transduction pathways that occur in ECs in response to LPS stimuli.

## LPS activates endothelial cells

EC activation is considered a key feature of the host defense response, but prolonged EC activation results in inflammatory dysregulation, endothelial injury, endothelial dysfunction, and the development of sepsis [55, 56]. Upon exposure to LPS, ECs undergo significant changes in function and gene expression, which are largely involved in immune responses, inflammatory aggravation, and thrombogenicity [57, 58]. As a biomarkers of EC activation, the levels of vWF and P-selectin were correlated with the levels of pro-inflammatory cytokines and chemokines [59]. In an in vitro experimental model of sepsis, there was progressive endothelial cell activation in correlation with the severity of sepsis, with significantly increased expression of surface adhesion receptors, such as ICAM-1, VCAM-1 and vWF, etc. [60]. Active Tie2maintains vascular quiescence, including barrier function. During sepsis, Tie2 activation leads to signaling inhibition, promoting vascular leakage, inflammation, and thrombosis [61]. Endothelial activation in sepsis leads to the expression of E- and P-selectin, VCAM-1, and ICAM-1 on the endothelial cell surface, which in turn causes leukocytes to roll, adhere, and transmigrate across the endothelial cells [62]. Therefore, there is a good biological rationale to use markers of endothelial activation as biomarkers of infection and inflammatory processes [63].

In the clinic, essential EC active molecules have been investigated as potential biomarkers for the early diagnosis, classification, and prognosis of sepsis [20, 64]. Many reliable biomarkers for monitoring EC activation have been established to address these issues. In a clinical study of patients with septic shock, circulating endoglin levels were significantly higher in patients with early mortality in septic shock. In addition, endoglin levels correlated with symptoms of circulatory failure [65]. Novel therapeutic strategies will be provided by numerous targets along the complex and redundant immune response pathways associated with ECs [54, 66]. The markers localize on the EC surface and interact with ligands to activate different signaling mechanisms, which can be classified into the following categories:

- Receptor and signal recognition systems. By sensing various proinflammatory and anti-inflammatory cytokines, exosomes and extracellular vesicles, ECs are able to recognize stimuli and danger signals.

(b) Activation and immune surveillance function of ECs. In response to stimuli, ECs can synthesize and secrete cytokines, chemokines, adhesion molecules, exosomes, etc., to regulate inflammatory events.

(c) Antigen presentation. At the response site, ECs have the ability to present antigens to adaptive immune cells, including B and T lymphocytes. Immune cell migration, exacerbation of inflammation, and platelet aggregation are modulated by the cooperation of ECs with the adaptive immune system.

(d) Cell‒cell interaction. The intimate interaction with ECs provides guidance to other cells, inducing them to become activated and differentiated. ECs are capable of regulating migrating immune cells, inflammatory cells, smooth muscle cells and others.

We believe that these biomarker changes in ECs will facilitate signal conduction studies and provide a more accurate perspective of the mechanisms driving EC activation. Biomarkers expressed on activated endothelial cells are summarized in Table 1.

**Table 1:** Biomarkers expressed on activated endothelial cells

Endothelial markers	Ligands	Effects
Endoglin	TGF-β	Essential for angiogenesis
vWF	Factor VIII, platelet glycoprotein, heparin, and collagen	Regulating angiogenesis, proliferation and migration as well as Ang-2 release
ICAM-1	ICAM-1	Modulating leukocyteadhesion and movement on endothelial cells
VCAM-1	VLA-4 integrin	Modulating leukocyteadhesion and movement on endothelial cells
PECAM-1	CD31	Promoting endothelial intercellular junction to enhance endothelial barrier properties. modulate leukocyte adhesion and movement on endothelial cells
E-selectin	Sialyl-Lewis X antigen and other carbohydrates	Modulating leukocyte movement on endothelial cells
P-selectin	Carbohydrate determinants on selectin ligands	Regulating leukocyte adhesion and movement on endothelial cells
VEGFR-1(Flt-1)	VEGF	Stimulating endothelial cell migration, hyperpermeability, and angiogenesis
VEGFR-2 (Flk-1)	VEGF	Promoting certain cancers progression
Tie-2	Angiopoietins	Regulating angiogenesis and permeability of endothelial cells
ACE	Angiotensin	Catalyzing the conversion of angiotensin I to angiotensin II to induce vasoconstriction and increase vascular permeability
ANGPTLs	Leukocyte immunoglobulin-like receptors	Regulating angiogenesis and some may exhibit other functions

*TGF-β* Transforming growth factor-β, *vWF* von Willebrand factor, *ICAM-1* Intercellular adhesion molecule-1, *VCAM-1* Vascular cell adhesion molecule-1, *PECAM-1* Platelet endothelial cell adhesion molecule-1, *VEGF* Vascular endothelial growth factor, *VEGFR* Vascular endothelial growth factor receptor, *Ang* Angiopoietins, *ANGPTL* Angiopoietin-like proteins, *Flt-1* Fms-like tyrosine kinase-1, *Flk-1* Fetal liver kinase-1, *ACE* Angiotensin converting enzyme, *eNOS* Endothelial nitric oxide synthases

## LPS induces endothelial injury

ECs detect microbial cues and then orchestrate the host immune response to defend against pathogens [67]. This crucial recognition task is usually performed by PRRs that can discriminate between self or microbe molecular structures [5]. Upon recognition of microbial substructures, such as LPS, ECs are able to activate intracellular signal transduction pathways that lead to the secretion of cytokines and the expression of cellular adhesion molecules and procoagulant substances [39, 68]. The immune significance of ECs is underlined by the recently proposed pathophysiological model of sepsis based on the approach of targeting pathogen-associated molecular patterns (PAMPs) and danger-associated molecular patterns (DAMPs) [69]. ECs can sense PAMPs and DAMPs to facilitate effective immune responses by activating and regulating immune cells [67, 70]. During early sepsis, PAMPs orchestrate the innate immune response through stimulation of PRRs, which trigger subcellular signaling pathways and upregulate the expression of a variety of proteins to resolve the host inflammatory response [64, 71]. ECs are capable of attracting different types of immune cells and are involved in cytokine secretion, phagocytic function, antigen presentation, and pro-inflammatory, pro-immune, anti-inflammatory, and immunosuppressive processes [72, 73]. When the host inflammatory response is exaggerated, a systemic inflammatory response develops that is essentially maladaptive, leading to tissue damage and organ dysfunction [74, 75]. These findings establish ECs as a potential drug target in the treatment of sepsis and other types of inflammatory diseases.

However, the development of sepsis has been linked to extensive endothelial damage and multiple apoptotic events involving direct endothelial dysfunction [58, 64]. Direct biomarkers of endothelial damage may be of great interest in sepsis. For example, adhesion molecules (ICAM-1, VCAM-1, E-selectin, and P-selectin) can be detected in the serum of septic patients and reflect endothelial cell activation (or endothelial dysfunction) and increased leukocyte–endothelial interactions [76, 77]. This may be an important prognostic marker for sepsis severity and prognostic value. In addition to sepsis, EC dysfunction has been reported in several other conditions with similar predictive values, including diabetes and cardiovascular and renal disorders [55, 78]. As mentioned above, excess proinflammatory cytokines, including PAMPs and DAMPs, are released as a result of subcellular signal transduction [79, 80]. After activation of PPRs, key cytokines such as TNF-α, IL-1β, and IL-6 are secreted. These cytokines amplify the production of inflammatory mediators, including CRP, IL-6, sFLT-1, angiopoietin-2, fibrinogen, von Willebrand factor, thrombomodulin, tissue plasminogen activator, surfactant proteins and SP-D, soluble receptors, and so on [81]. During sepsis, these inflammatory mediators exert deleterious effects on ECs, including disassembly of intracellular junctions, alteration of cytoskeletal structure, or damage to the cell monolayer, usually resulting in microvascular leakage and tissue edema [55]. For instance, sFlt-1 shows promise as a novel biomarker of sepsis severity with the strongest association with Sequential Organ Failure Assessment score [60]. Angiopoietin-2 level is proportional to the severity of the disease, continues to increase over time, and is predictive of the future occurrence of shock or death [82]. In addition, endothelial glycocalyx breakdown in human sepsis is mediated via Tie2 deactivation by angiopoietin-2. Activation of Tie2 seems to accelerate recovery of the eGC and might hold promise as a therapeutic target in human sepsis [83]. Furthermore, ECs increase vascular wall expression of intracellular adhesion molecule (ICAM), vascular cell adhesion protein-1 (VCAM-1), and platelet–endothelium cell adhesion molecule (PECAM) [68, 84]. The sepsis-induced procoagulant phenotype of ECs is characterized by increased production of tissue factor (TF) and subsequent activation of the extrinsic coagulation pathway [62]. Several pathways have been proposed to be involved in the pathophysiology of endothelial dysfunction in COVID virus infection [66, 85]. Therefore, different pathways of endothelial dysfunction may be involved, and different therapies that target one or more of these pathways may be useful in treating sepsis. Biomarkers of endothelial injury are summarized in Table 2.

**Table 2:** Biomarkers of endothelial injury

Markers	Effects	Clinical implications
Occludin	Occludin is a major component of the tight junction of epithelial and/or endothelial barriers	Occludin level is increased in numerous pathologic conditions, including HIV, cancer, neuroinflammation and sepsis
Syndecan-1	Syndecan-1 is released in endothelial glycocalyx damage	Syndecan-1 levels in circulation are associated with severity of sepsis, acute kidney injury, need for intubation, and mortality
Endocan	Endocan is a soluble dermatan sulfate proteoglycan in endothelial cells	Endocan can regulate major processes, such as cell adhesion, in inflammatory disorders and tumor progression
claudin-5	Claudin-5 can modulate the permeability of tight junctions	Levels of claudin-5 in serum is to correlate with severe plasma leakage
cadherin-5	Cadherin-5 is a main component of adherens junction	Loss of vascular endothelial cadherin results in increased permeability
ZO-1	ZO-1 is a component of tight junction proteins	ZO-1 is associated with inflammation and cancer
TF	Exposure of TF attract interaction with the FVII and FX that activate both coagulation cascades	TF can activate both coagulation cascades and increase vascular permeability
PAI-1	As the main negative regulator of plasminogen activation, PAI-1 is an essential factor in regulation of the physiological balance between thrombosis and fibrinolysis	High PAI-1 levels are associated with many cardiovascular diseases
Antithrombin	Antithrombin is the active anticoagulant operative during heparin therapy to inhibit thrombin, factor 10a, and, less efficiently, factors 9a and 11a	Heparin-like molecules are synthesized by endothelial cells and interact with AT on the vessel wall to inhibit coagulation
Thrombomodulin	Thrombomodulin is an endothelial transmembrane glycoprotein that possess a central modulatory role in the natural anticoagulant system	Thrombomodulin can blocks thrombin to inactivate the pro-coagulant signaling and the downstream pro-inflammatory responses
Angiopoietin-1/2	Angiopoietin-1 and 2 (Ang1, Ang2) are important mediators of angiogenesis	Perturbation of Angiopoietin-1/2 leads to various pathological conditions, such as inflammation, tumor and restenosis
sICAM-1	sICAM-1 can mediate leukocyte migration and adhesion to target structures by binding to the leukocyte adhesion receptor	sICAM-1 is upregulated in endothelial dysfunction and promotes an inflammatory response
sVCAM-1	sVCAM-1 plays an important role in the adhesion and migration of leukocytes from blood to vascular intima	sVCAM-1 are considered to be markers of endothelial cell activity or injury
sFlt-1	sFlt-1 is a soluble antagonist of VEGF with an essential effect of maintaining the balance of vascular growth	sFlt-1 is associated with markers of inflammation, endothelial function, and myocardial stress or injury
sE-Selectin	E-selectin is a recognized marker of endothelial activation	sE-selectin expression by endothelial cells is crucial for leukocyte recruitment during the inflammatory response

*ZO-1* Zonula occludens-1, *TF* Tissue factor, *PAI-1* plasminogen activator inhibitor-1, *sICAM-1* soluble intercellular adhesion molecule-1, *sVCAM-1* soluble vascular cell adhesion molecule-1, *sFlt-1* soluble fms-like tyrosine kinase-1, *sE-Selectin* soluble E-selectin

## LPS deteriorates endothelial permeability

Intriguingly, TLR activation also modulates microvascular endothelial cell permeability [86]. During infection, LPS activates endothelial signaling pathways to secrete various proinflammatory mediators, such as TNF-α, IL-6, IL-8, and IL-1β [87, 88]. These mediators subsequently induce the secretion of chemokines and adhesion molecules and decrease anti-inflammatory mediators, leukocyte transmigration, and the production of reactive oxygen species [89]. Increased production of inflammatory cytokines disrupts the arrangement in the endothelial barrier. High expression of ICAM-1 and VCAM-1 molecules induces an immune response in ECs [90, 91]. LPS also strongly induces the expression of key complement factors, such as C1, C3, and C5, in accordance with the current role of their involvement in host lung defense and endothelial damage during sepsis-associated acute lung injury [92]. A significant change in endothelial permeability after exposure to LPS-stimulated leukocytes has been confirmed. The deleterious effect of LPS on lung cells is due to disruption of the alveolar capillary barrier in early sepsis [17, 93]. Sepsis is associated with early and profound endothelial glycocalyx injury, and circulating endothelial glycocalyx components are directly correlated with clinical severity and outcome [94]. Furthermore, deterioration or reduction of the endothelial glycocalyx is associated with subsequent pathophysiology, including increased endothelial permeability, platelet aggregation, coagulopathy, and loss of vascular responsiveness. The protective effects associated with the glycocalyx are manifested as enhancement of endothelial barrier function, prevention of intravascular coagulation, attenuation of leukocyte adhesion, and induction of NO release [95]. Thus, impaired endothelial permeability is one of the major pathological features of sepsis.

Shear stress and pulsatile shear stress generated by the mechanical frictional forces of blood flow and cardiac contraction are well-known mechanical signals that promote EC homeostasis and cardiovascular health [96]. Thus, fluid shear stress, the tangential frictional force exerted by flowing blood, also influences endothelial permeability [97]. During sepsis, decreases in blood flow velocity or changes in flow pattern are associated with decreased NO production and may exacerbate leukocyte adhesion, platelet aggregation, and inflammation [98]. Vascular endothelial growth factor (VEGF) is an important regulator of vascular permeability through the regulation of nitric oxide synthase (NOS) [99]. Various inflammatory or infectious factors activate membrane calcium channels on ECs and promote calcium influx across the membrane, which induces the formation of interendothelial cell gaps and EC hyperpermeability, leading to increased endothelial permeability [100]. Taken together, infectious and pathological stimuli can cause acute and dramatic changes in endothelial permeability. As research continues to delve deeper into the molecular mechanisms, these findings will provide new ideas and new methods to maintain endothelial permeability.

## LPS contributes to intravascular coagulopathy

By balancing coagulant and anticoagulant properties, ECs play a key role in maintaining intravascular patency and permeability [57]. Sepsis-associated coagulopathy is the result of inflammation-induced activation of intravascular coagulation pathways accompanied by dysfunction of the anticoagulant and fibrinolytic systems, leading to varying degrees of hemostasis dysregulation [101, 102]. LPS is one of the most predominant pathogenic factors inducing hypercoagulability or hypocoagulability. LPS binds to TLR4 to activate ECs and platelets, thereby initiating the coagulation cascade [103, 104]. Coagulation activation can be induced not only by the microbiome but also by other important pathways, including PAMPs and DAMPs, neutrophil extracellular traps, extracellular vesicles, and glycocalyx damage [101, 105]. This hematologic response is initially beneficial and contributes to the clearance of the microbiome, but the loss of control of coagulation activation leads to widespread microvascular thrombosis, the development of sepsis-induced disseminated intravascular coagulation (DIC), and subsequent organ failure.

**Fig. 3:**
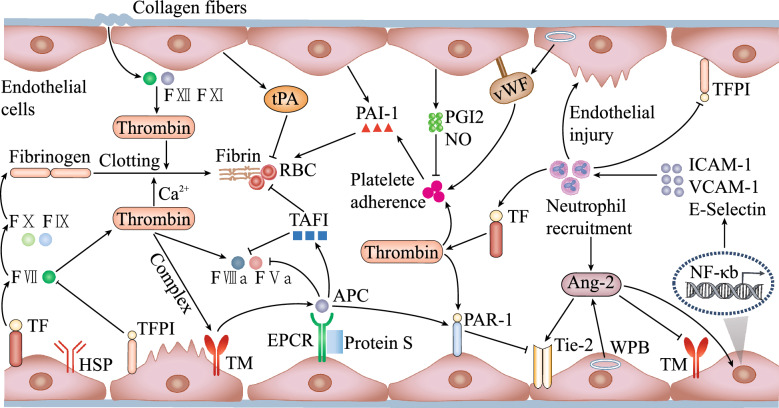
Mechanisms of intravascular coagulation in sepsis. Sepsis-induced coagulopathy involves both intrinsic and extrinsic coagulation pathways. After vessel damage, the fibers directly activate plasma clotting factor XI (FXI). Injury to the vascular wall also allows FVIIa to come into contact with fibroblasts that carry the TF receptor. The FVIIa/TF complex then activates FX and FIX. Thrombin is generated, and FV, FVIII, FXI and thrombocytes are activated. This pathway serves to propagate coagulation, resulting in the generation of large amounts of thrombin and fibrin. When thrombin binds to the endothelial cell surface protein thrombomodulin (TM), the substrate specificity of the enzyme is altered, resulting in loss of its procoagulant activity. The thrombin–TM complex acts as an anticoagulant by activating protein C (PC) and subsequent thrombin-activatable fibrinolysis inhibitor (TAFI), which attenuates the coagulation cascade by inactivating activated cofactors V and VIII. Endothelial cells have heparan sulfate proteoglycans that bind and enhance plasma coagulation proteins, including tissue factor pathway inhibitor (TFPI) and antithrombin (AT) (synthesized in the liver). Thrombomodulin (TM), which promotes thrombin-mediated activation of protein C (PC), is also expressed on endothelial cells. This reaction is amplified by the endothelial protein C receptor (EPCR). Activated protein C (APC) inactivates FVa and FVIIIa, thereby limiting coagulation. Endothelial cells synthesize and release tissue-type plasminogen activator (tPA), which promotes plasminogen (Plg) to plasmin (Pln) conversion. TM/thrombin interacts with the coagulation–fibrolysis system to act as a negative feedback loop in the presence of APC–EPCR. The interaction between APC and EPCR also enables the switching of the PAR-1 signal to an anti-inflammatory pattern and enhances the integrity of the endothelium via S1P- and Ang/Tie-mediated activities. Fibrinolytic inhibitors, such as plasminogen activator inhibitor-1 (PAI-1) and thrombin-activatable fibrinolytic inhibitor (TAFI), are upregulated under septic conditions and aggravate microvascular thrombosis by preventing fibrin degradation. PAI-1 is produced by ECs, megakaryocytes, smooth muscle cells, fibroblasts, monocytes, adipocytes, hepatocytes, and other cell types. Platelets store a pool of PAI-1, which accounts for more than half of its availability and helps deliver it to the clot. TAFI circulates in the plasma and may be activated by the thrombin-thrombomodulin complex. Thrombin-induced conversion of TAFI to activated TAFI (TAFIa) supports the important role of the coagulation cascade in regulating fibrinolysis. In the context of sepsis, the stimulation of inflammatory cytokines, coagulation factors or VEGF allows the secretion of Ang2 from the WPB. Through an autocrine loop mechanism, Ang2 itself acts as a fast-acting regulator of the endothelium. Thrombomodulin is an endothelial cell surface molecule that plays an essential role as an anticoagulant by acting as a cofactor in the thrombin-mediated activation of protein C and thrombin-activated fibrinolysin inhibitor (TAFI). Ang-1 and Ang-2 are effective in inhibiting the generation of activated protein C and TAFI by thrombin and TM in cultured endothelial cells and in inhibiting the binding of thrombin to TM in vitro. Ang2 appears to be a more potent inhibitor of TM function, binding to TM with higher affinity than Ang1. **↑**: activation, ┫: inhibition

In fact, in the host coagulation system, ECs play dual roles. That is, they participate in the process of clot formation and prevention of thromboembolism, maintaining blood fluidity based on the homeostatic state [106]. Under normal conditions, the defense function of the coagulation system is maintained, and excessive coagulation activation is avoided [107]. In sepsis, however, the anticoagulant properties of ECs are severely impaired. Simultaneously, activated or injured ECs can provide scaffolds for intravascular clotting [86]. For example, tissue factor (TF) expression is a key initiator of the procoagulant pathway and is induced on the surface of activated ECs. Phosphatidylserine, another high affinity framework for clotting factors, including FII, FVII, FIX, and FX, is externalized to the plasma membrane of injured ECs [57, 108]. Induction of TF can induce thrombin generation on the surface of LPS-activated ECs, whereas treatment with anti-TF antibodies completely prevents thrombin generation on LPS-pretreated ECs [57]. The role of both inflammatory pathways and coagulopathy due to endothelial dysfunction has been extensively studied in sepsis [109]. It is conceivable that activated or injured ECs could provide both initiating and inhibiting factors of the coagulation pathway, although the latter is always insufficient compared to normal ECs. The dual role of ECs in addressing the coagulatory/anticoagulant balance is illustrated in Fig. 3.

DIC is known to be a lethal complication of sepsis, and its early recognition and appropriate control of the underlying infection are the current effective management strategies [110, 111]. Treatment with recombinant antithrombin and thrombomodulin can partially suppress aberrant activation of coagulation pathways [57]. However, under septic conditions, intravascular anticoagulant potential may be compromised by downregulating endothelial thrombomodulin, disrupting the endothelial glycocalyx, and reducing plasma anticoagulant properties, such as TFPI and antithrombin [57, 112]. In addition, the development of overt DIC is associated with the loss of endogenous anticoagulant protein C and an increase in the vascular regulator angiotensin-2 [113]. In the population with sepsis-induced DIC, clinical trials have suggested a survival benefit with anticoagulant therapy. In septic patients with DIC, a reduction in 28-day mortality has been observed with the use of anti-thrombin therapy [114]. Large clinical trials of anticoagulation in sepsis have not shown a survival advantage, but database analyses and several smaller studies have shown beneficial effects of anticoagulation in subpopulations of patients with early sepsis-related disseminated intravascular coagulation [8]. These observations suggest that there is a favorable prospect of intervention with anticoagulant therapy in the population of patients with sepsis-induced DIC.

However, concerns have been raised. The current cutoff points of the DIC scoring systems may be suboptimal for the determination of the severity of the disease and may delay the initiation of anticoagulant therapy [115]. The point at which coagulation activation switches from beneficial to detrimental indicates a reasonable opportunity for the administration of targeted anticoagulant therapy [116]. To date, the optimal anticoagulant therapy in septic patients is not well-established. The inclusion criteria for appropriate patients and the appropriate duration of treatment need to be determined.

## LPS impels immunoparalysis

During the late stage of sepsis, patients are profoundly immunosuppressed because of the abundant apoptosis and tolerance of immune cells [117, 118]. Cytosolic LPS-induced pyroptosis is the main driver of endotoxic shock, highlighting the pivotal role of caspase-4/5/11 as intracellular LPS receptors [119]. Thus, LPS-induced EC pyroptosis via inflammasome activation contributes to immunosuppression in later stages of sepsis [51]. Nevertheless, sepsis-induced immunoparalysis can be quite heterogeneous. That is, the extent and nature of the underlying immune defects vary significantly between patients and within a single patient over time [120]. Therefore, several antimicrobial and antiendotoxin agents are known to conjugate with LPS and have been used to block LPS-induced activation of TLR4 [121, 122]. However, such a strategy is hampered by the lack of currently available indicators to identify the various immune defects that may manifest differently in individual patients [123]. Furthermore, due to their mechanism of action, these agents should be able to inhibit cytosolic LPS binding to its intracellular receptor to protect ECs from apoptosis and pyroptosis. Therefore, while a 'one-treatment-fits-all' strategy for sepsis-induced immunoparalysis has not been established, an individualized 'precision medicine combined with targeted treatments' approach is needed.

## Treatments for LPS-induced sepsis

Sepsis continues to be the most important cause of morbidity and mortality in critical care patients. Several steps may be involved in the development of LPS-induced sepsis: (a) microbial invasion, (b) recognition of bacterial products (e.g., lipopolysaccharide), (c) immune response and immune dysregulation, (d) endothelial and organ damage, and (e) organ crosstalk and multiple organ dysfunction. Each of these steps can be a potential target for a specific therapeutic approach. At various stages, extracorporeal therapies may be a target for the removal of circulating molecules. Therefore, the following may be consideration of:

### (a) LPS vaccine

Against certain pathogens, a specific immune response to LPS molecules can induce protective immunity. Current antibiotics remain unsatisfactory due to poor targeting efficiency and poor drug penetration through the bacterial cell wall. Thus, targeted delivery of antibiotics into gram-negative bacteria should be a promising approach [124]. For these reasons, purified derivatives of LPS could be used as a parenteral vaccine. Modifying the structure of LPS makes it possible to induce the proper immune response required in a vaccine against a specific pathogen while reducing toxicity [125]. Studies have shown that LPS with a mutation in the glycosyltransferase WadC is more efficiently recognized by MD2. This leads to an increased cytokine response. Lipid A-free LPS is also a suitable immunogen in mice [126]. Here, LPS is a natural adjuvant with tunable properties to guide the immune response. Therefore, this glycolipid is an ideal target for developing live attenuated gram-negative vaccines [8, 127]. However, potential risks, such as virulence in susceptible hosts and potential reversal of attenuation, may remain with live attenuated vaccines that induce a strong immune response against bacterial pathogens. Although there is still much work to be done due to the diversity of LPS in different bacterial species, it is likely that this molecule will become one of the most important targets for future vaccine development.

### (b) LPS antagonist

Synthetic anti-LPS peptides are designed to bind to LPS and LP. Based on the inhibition of the inflammatory effect of LPS, they can inhibit inflammation regardless of the resistance status of the bacteria [128, 129]. Studies have shown that both vitexin and donepezil are able to bind in close proximity to the LPS binding site located on the TLR4-MD-2 complex, which has the potential to be a candidate antagonist for LPS [130]. TLR4 antagonist treatment significantly increases survival, ameliorates lung necrosis, and inhibits inflammatory cytokine secretion in septic shock mice [131]. These studies point to the promise of the therapeutic effects of LPS antagonists in the treatment of sepsis.

### (c) LPS removal

For the control of both infection and hyperinflammation, LPS removal is beneficial. Simultaneous adsorption of septic molecules such as LPS, cytokines and DAMPs/PAMPs from blood with high efficiency is achieved by immobilization of telodendrimer nanotraps [132]. Another novel antimicrobial platform, composed of a mesoporous copper–silicate microsphere core and a platelet membrane shell, exhibits robust antimicrobial activity and strong toxin adsorptivity, which facilitates the clinical treatment of many bacterial infections and the development of next-generation antimicrobial nanoagents [133]. It also shows great promise for the neutralization of LPS and simultaneous delivery of antibiotics. A polymyxin B-modified liposomal system can target *E. coli* by binding to LPS and synergistically adsorbing free LPS, thereby promoting infection control [124]. In addition, experiments in mice show that injection of a water-soluble flexible organic scaffold at an appropriate dose improves the survival of mice administered a lethal dose of LPS [134]. In our opinion, the removal of LPS from the circulation with affinity binders may be a novel approach to the delivery of antibacterial agents for the treatment of persistent and severe bacterial infections.

### (d) Cytokine removal

Extracorporeal cytokine removal technology, designed to reduce the cytokine storm in inflammation, is already used as an early standalone therapy to treat inflammation in critically ill patients. Treatment with etanercept, a TNF-α antagonist, significantly attenuated LPS-induced inflammation in the systemic circulation [135]. Cannabidiol attenuated both LPS-induced cytokine release and NF-κB activity in vitro [136]. In addition, emodin attenuated LPS-induced levels of inflammatory cytokines and cardiac inflammation in mice [137]. MCC950, a specific NLRP3 inhibitor, is effective in inhibiting LPS-induced lung inflammation in vivo and may be considered for clinical translation [138]. Taken together, these findings suggest that the protective effect of cytokine depletion by attenuating the inflammatory response and inhibiting the activation of the NLRP3 inflammasome may provide a viable strategy for the prevention and treatment of organ injury in sepsis.

### (e) Extracorporeal organ support

It has been recognized that organ failure does not occur in isolation but rather results from, and has an impact on, the dysfunction of other organs, mediated by an interplay that has been termed organ crosstalk [139]. Various techniques for respiratory, cardiac, and renal support have significantly improved outcomes in sepsis patients [140]. Mechanical ventilation is a cornerstone of critical care and one of the most commonly used life support interventions in critically ill patients [141]. Venoarterial extracorporeal membrane oxygenation (ECMO) provides both pulmonary and circulatory support to critically ill hemodynamically compromised patients as a bridge to recovery or definitive therapy in the form of transplantation or permanent ventricular support [142, 143]. Renal support (hemofiltration, hemodialysis or ultrafiltration) has been proposed as a promising adjuvant therapy for the treatment of critically ill patients by removal of cytokines and DAMPs/PAMPs from the blood [144]. Continuous renal replacement therapy (CRRT) is now the dominant form of renal replacement therapy in the ICU due to its accurate volume control, continuous acid‒base and electrolyte correction, and achievement of hemodynamic stability [145]. However, sequencing and combining different extracorporeal therapies to achieve specific goals should be optimized. We hypothesize that these therapeutics may share common mechanisms.

## A new therapeutic paradigm for sepsis

There is increasing evidence that the diversity of endothelial function is essential for the maintenance of vascular homeostasis and that endothelial dysfunction is a characteristic feature of a broad spectrum of vascular diseases involving vasoconstrictive, thrombogenic, and inflammatory pathologies [146]. Molecular biological research has shown that ECs synthesize and release a variety of endothelium-derived relaxing factors, including vasodilator prostaglandins, nitric oxide, and endothelium-derived hyperpolarizing factors, as well as endothelium-derived contracting factors that mediate vascular tone [147]. Despite appropriate source control and antibiotic coverage, there are no specific therapies to treat sepsis, and alternative treatment strategies are being explored to prevent complications and improve outcomes.

A hallmark of the pathophysiology of sepsis is microvascular dysfunction. Binding of LPS to the surface of ECs directly induces endothelial activation, as evidenced by increased levels of proinflammatory cytokines and adhesion molecules and, in some cases, endothelial injury and apoptosis [93, 148]. In addition, LPS stimulates other immune cells, such as monocytes and macrophages, to express inflammatory mediators that affect endothelial function [57, 149]. Thus, LPS initiates a parallel cascade of immune responses that manifest as the complex clinical manifestations of sepsis. Furthermore, the resulting endothelial dysfunction is thought to contribute to the underlying pathogenesis of sepsis and organ dysfunction [150]. Thus, endothelial dysfunction is central to sepsis pathogenesis, including exaggerated inflammation, coagulation, vascular leakage and tissue hypoperfusion [151, 152]. In contrast, activation of inflammatory, coagulation, and other pathways are fundamental host responses to infection but also cause injury to host tissues. Modulation of endothelial function has great potential for the development of therapeutics to treat sepsis, since ECs are involved in both the immune response to infection and the pathological responses in sepsis. Much work has already explored the utility of targeting different endothelial pathways for the diagnosis and treatment of sepsis. TRIM47, an E3 ubiquitin ligase, has recently been identified as a novel activator of endothelial cells. It promotes LPS-induced pulmonary inflammation and acute lung injury and activates NF-κB and MAPK signaling pathways to induce an inflammatory response in endothelial cells [153]. Vasopressin, IFN-β, and thrombomodulin are considered potential therapeutic agents with endothelial cell protection properties [154]. Inhibition of *E. coli* binding to the endothelial cell integrin αVβ3 by cilengitide prevents endothelial dysfunction. Therefore, cilengitide may represent a novel early therapeutic option for the treatment of sepsis [155]. Interestingly, inhibition of PFKFB3 alone or in combination has also shown great potential in the treatment of sepsis [156]. Therefore, combined strategies for the prevention of endothelial dysfunction are promising for the treatment of sepsis.

## Conclusions

LPS is one of the key triggers of lethal sepsis and is the most extensively studied and recognized microbial component as a primary driver of the cytokine storm. Multiple pathways, both extracellular and intracellular, are involved in the sensing of LPS, and the subsequent immune response is thought to play a major role in the pathophysiology of sepsis. Although research has tremendously advanced the pathophysiology of sepsis, this complex syndrome remains incompletely understood. The severity and mortality of sepsis are associated with endothelial injury and dysfunction. Thus, reducing the magnitude and severity of complications resulting from endothelial dysfunction may be a major benefit of further developing and advancing therapeutic strategies to prevent or minimize endothelial injury. Important areas for future research are expected to include improvement of endothelial cell function to provide endothelial protection and reduce edema formation, blood purification techniques to restore immune homeostasis, and immunostimulation therapy for immunocompromised patients.

## Data Availability

Not applicable.
